# Augmented Reality-Assisted Transcanal Endoscopic Ear Surgery for Middle Ear Cholesteatoma

**DOI:** 10.3390/jcm13061780

**Published:** 2024-03-20

**Authors:** Keisuke Tsuchida, Masahiro Takahashi, Takara Nakazawa, Sho Kurihara, Kazuhisa Yamamoto, Yutaka Yamamoto, Hiromi Kojima

**Affiliations:** Department of Otorhinolaryngology, Jikei University School of Medicine, Minato-ku, Tokyo 105-8461, Japan; k.tsuchida@jikei.ac.jp (K.T.);

**Keywords:** augmented reality, cholesteatoma, tympanoplasty, transcanal endoscopic ear surgery (TEES)

## Abstract

**Background:** The indications for transcanal endoscopic ear surgery (TEES) for middle ear cholesteatoma have expanded for cases involving mastoid extension. However, TEES is not indicated for all cases with mastoid extension. In addition, predicting the extent of external auditory canal (EAC) removal needed for cholesteatoma resection is not always easy. The purpose of this study was to use augmented reality (AR) to project the lesion onto an intraoperative endoscopic image to predict EAC removal requirements and select an appropriate surgical approach. **Methods:** In this study, patients showing mastoid extension were operated on using a navigation system with an AR function (Stryker). **Results:** The results showed that some cases with lesions slightly extending into the antrum required extensive resection of the EAC, while cases with lesions extending throughout the antrum required smaller resection of the EAC, indicating TEES. **Conclusions:** By predicting the extent of the needed EAC removal, it is possible to determine whether TEES (a retrograde approach) or canal wall-up mastoidectomy, which preserves as much of the EAC as possible, should be performed. We believe that our findings will contribute to the success of middle ear surgeries and the implementation of robotic surgery in the future.

## 1. Introduction

Cholesteatomas are benign collections of keratinized squamous epithelium, mostly found within the middle ear; however, they are an intractable chronic proliferative disease that can cause fatal complications, such as bone destruction and brain abscesses. The only treatment option is surgery, and the long-term recurrence rate is reported to be as high as approximately 20–60% [1,2,3,4,5,6,7]. Recurrence rates vary widely depending on the method of statistical analysis and period of observation, making it difficult to determine the optimal strategy for selecting a procedure. Nevertheless, the goal should be to ensure all lesions are removed and reconstructed while preserving as much of the normal structure as possible. The extent of the lesion, particularly its extension into the mastoid, affects the choice of the surgical approach, such as transcanal endoscopic ear surgery (TEES) or microscopic ear surgery (MES) with a postauricular incision [8]. TEES, which does not require a postauricular incision, is less invasive [9] but is not indicated for all cases. The external auditory canal (EAC) is often missing when TEES is applied in cases with mastoid extension because the EAC is drilled to approach the lesion in TEES. Excessive drilling can make ear canal reconstruction difficult and lead to an incomplete postoperative state. The ideal approach is to resect the lesion while preserving the ear canal using TEES and to ensure reconstruction of the defect. We have actively used a 70°endoscope in TEES to reduce excessive EAC drilling [10,11]. In cases that require extensive EAC drilling, even with an angled endoscope, MES, a transcortical approach in which the EAC can be preserved (canal wall-up mastoidectomy), should be applied. Imaging examinations, such as computed tomography (CT) and magnetic resonance imaging (MRI), are used to predict the extent of progress and select a surgical approach. However, this decision is not easy to make, and sometimes unintentional excessive drilling or an intraoperative change of approach is necessary, leading to prolonged operative time [11]. Therefore, we focused on using augmented reality (AR) to make optimal intraoperative decisions. AR is a generic term for advanced technologies that superimpose computer-generated images onto scenery that can be viewed with the naked eye. The term AR was first defined in 1992 [12], and AR technology applications date back to the early 1900s, with the first optical devices used to assist military personnel in shooting [13]. The AR technology currently used in the medical field is associated with navigation systems, and its basic principle is to overlay preoperative images with a conventional endoscopic view [14,15,16,17]. In addition to projecting onto endoscopic images, AR glasses can be used to project information into the clinician’s field of vision. This has already been applied in spine, hip, and skull base surgeries [18,19,20]. In otologic surgery, there have been reports of cochlear implantation using AR with robotic assistance for cadavers [21] and preoperative AR projection in lateral temporal bone surgery to confirm the location of critical structures [22]. These are all types of MES, and to date, there are no reports on the use of AR in TEES. In TEES, AR can be used more simply and practically than in MES because AR can be projected directly onto the endoscopic image. Therefore, we aimed to investigate the possibility of using AR to determine the appropriate range for EAC drilling by projecting a lesion onto an intraoperative endoscopic image. The first aim of this study was to determine whether AR lesion projection is practical for TEES. The second aim was to determine whether AR allows the surgeon to select an appropriate technique. For example, the surgeon may continue with TEES or switch to a transcortical approach with MES.

## 2. Materials and Methods

### 2.1. Cases and Treatment Strategies

Cases in which mastoid extension was anticipated and could be considered an indication for TEES were included, while cases with very extensive extension and no obvious indication for TEES were excluded. We performed tympanoplasty using AR in five cases of cholesteatoma from January 2023 to September 2023. Our surgical treatment strategy for cholesteatomas with mastoid extension was as follows: TEES was indicated if the cholesteatoma could be removed by drilling the lateral wall of the attic using an angled endoscope. If the lesion was extensive and wide drilling of the posterior wall of the EAC was necessary, MES with a posterior ear incision was performed to preserve the posterior wall of the EAC. In both techniques, the ear canal defect was reconstructed using cartilage.

### 2.2. Navigation Systems

The navigation system used in this study is specialized for use in the field of otorhinolaryngology and is an electromagnetic tracking system (ENT navigation^TM^, Striker, Kalamazoo, MI, USA). AR images can be used by the software built into this machine. Rigid endoscopes with angles of 0°, 30°, 45°, or 70° (length: 14 cm, outer diameter: 3.1 mm; Stryker, Kalamazoo, MI, USA) for the transcanal endoscopic approach and a microscope (Zeiss OPMI Pentero 800, Carl Zeiss, Jena, Germany) for the transcortical approach were used. The endoscopes were connected to a camera head (Karl Storz Spies Image1 System TC200/TC300/TH100, Karl Storz, Tuttlingen, Germany), and a high-definition monitor was positioned in front of the surgeon.

### 2.3. Setup

**Figure 1:**
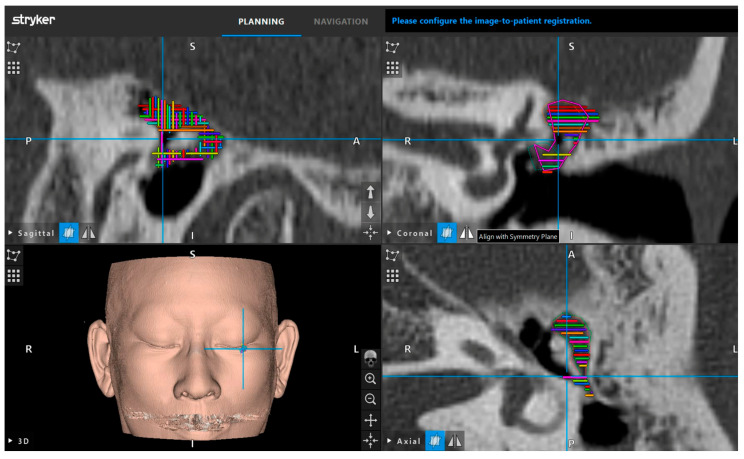
Navigation monitor after completion of tracing of lesion. The lesion was traced every 1.2 mm in three computed tomography (CT) directions and was ready for the augmented reality (AR) display after pre-planning was performed. The upper-left, upper-right, and lower-right images are sagittal, coronal, and axial slices, respectively. The tracing colors are freely selectable, and a variety of colors were used in this case.

All patients underwent CT of the temporal bone, while MRI was performed if the extent of the lesion could not be determined by CT. Lesions were marked every 1.2 mm using CT in three directions for AR imaging (Figure 1). CT without contrast was performed using a 64-MDCT scanner (SOMATOM Perspective; Siemens AG, Munich, Germany). The scanning parameters were as follows: collimation, 64 × 0.6 mm; rotation time, 1.0 s; detector-row width, 0.6 mm; pitch, 1.0; and scanning field of view (FOV), 25 cm. The peak tube voltage was maintained at 130 kV. The reconstruction parameters were as follows: section thickness, 0.6 mm, and 0.6 mm reconstruction in the axial plane. The CT threshold was adjusted using the bone algorithm (window center and width were fixed at 700 and 4000, respectively).

### 2.4. Registration

The field generator was placed on the cephalic side of the parietal lobe, and a reference marker was attached to the midline of the forehead to use relative coordinates. Registration was performed by tracing the facial skin using the pointer instruments provided with the navigation system (surface matching registration). Because the error increases with depth in surface matching registration, the malleus was used as a guide for correction (paired point registration). After completion of registration, the endoscope served as a navigation pointer by attaching instrument clamps to the endoscope. This made it possible to project AR images onto the endoscope screen.

### 2.5. Questionnaires

Ten otolaryngologists who had 5 to 33 years of experience (average 15.7 years) specializing in otologic surgery at our hospital and who utilized this strategy were questioned regarding their decision to use or not to use TEES in these cases on the basis of imaging findings, and they were also asked to show how they made the decision by presenting AR images. Four options were considered, including TEES without hesitation, probably TEES, probably MES, and MES without hesitation.

### 2.6. Statistical Analysis

Statistical analysis was performed using a statistical software package (JMP version 13; JMP Pro 14.0.0; SAS Institute Japan, Tokyo, Japan), and the Wilcoxon signed-rank test with continuity correction was used. A *p*-value of less than 0.05 denoted the presence of a statistically significant difference.

## 3. Results

**Figure 2:**
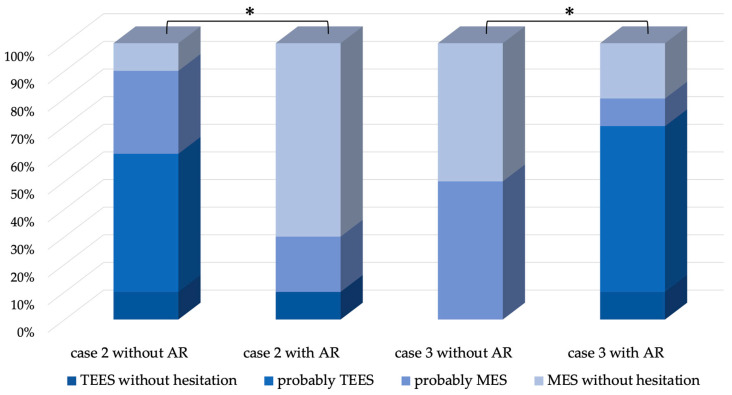
Questionnaire results of the two cases (Cases 2 and 3) in which the planned approach changed based on the AR display. The surgical approach in Case 2 and Case 3 changed significantly due to the AR display (*: *p* < 0.05). In Case 2, the proportion of performing MES increased significantly, while in Case 3, the proportion of performing TEES increased significantly. AR: augmented reality; MES: microscopic ear surgery; TEES: transcanal endoscopic ear surgery.

**Figure 3:**
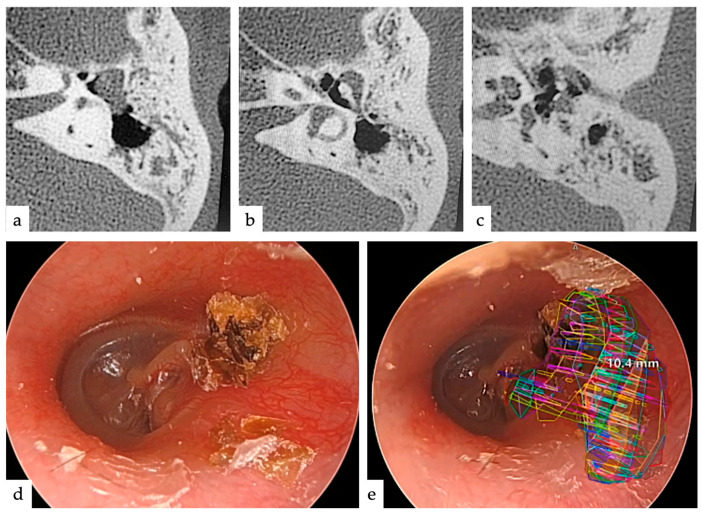
Imaging findings and AR display in Case 2. The AR display shows that the area of EAC drilling to be performed is large, whereas the area of progress is not very large. (**a**) Axial CT slice of the temporal bone showing a slight extension into the antrum. (**b**) Axial CT slice of the temporal bone showing extension into the attic. (**c**) Axial CT slice of the temporal bone showing extension into the tympanic cavity. The lesion extended close to the stapes; however, the superstructure did not exhibit any erosive changes. (**d**) Typical endoscopic findings of cholesteatoma with debris in the pars flaccida. (**e**) Endoscopic image with AR display overlaid on image (**d**). Extensive EAC drilling was required to remove the lesions. AR: augmented reality; EAC: external auditory canal; CT: computed tomography.

**Figure 4:**
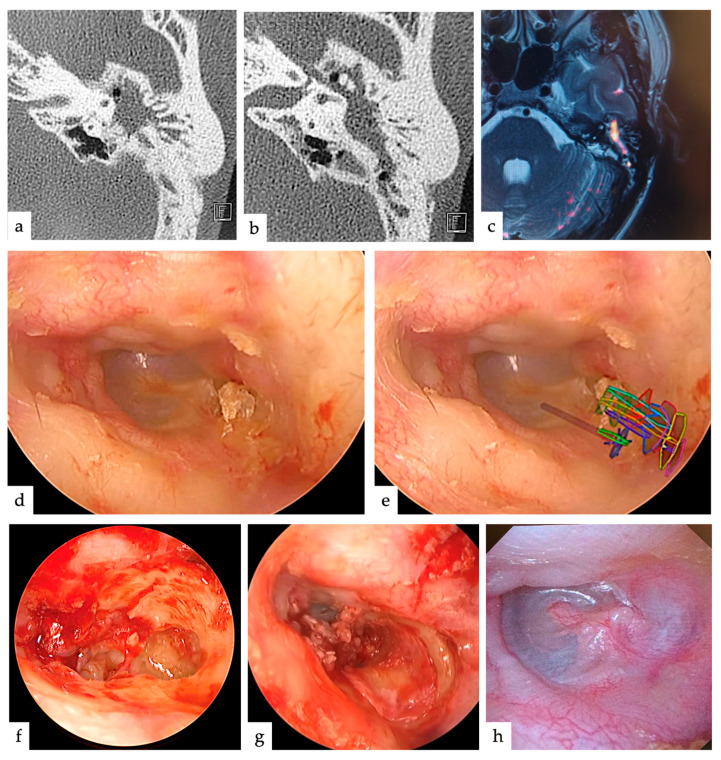
Imaging findings and AR display in Case 3. The AR display shows that the area of the EAC drilling to be performed is not very large, whereas the area of progress was relatively large. (**a**) Axial CT slice of the temporal bone showing extension into the entire antrum. (**b**) Axial CT slice of the temporal bone showing extension from the attic to the entire antrum. (**c**) MRI (non-echoplanar imaging (EPI)-based diffusion-weighted imaging (DWI) fused with T2) showing extension from the attic to the entire antrum, as well as (**b**). (**d**) Endoscopic findings of cholesteatoma with debris in the pars flaccida. Multiple mild osteomas were also observed in the EAC. (**e**) Endoscopic image with AR display overlaid on image (**d**). Extensive EAC drilling was not required to remove the lesion. (**f**) Endoscopic image after lesion removal. The range of the EAC drilling was consistent with that on the AR image. (**g**) The defect is covered with a single piece of tragus cartilage. (**h**) Otological findings 1 year postoperatively do not show retraction pockets.

The five patients who underwent surgery were aged between 5 and 51 years, including two males and three females, and the type of cholesteatoma was pars flaccida in four and congenital in one. All cases were operated on without complications, including four treated with TEES and one treated with MES. In all cases, AR images were projected onto the endoscopic image, and the planned approach was not changed during surgery. The mean operative time was 176.2 min, which did not change significantly with the use of AR. The extent of lesion extension identified during surgery was from the attic to the mastoid in three patients, and further to the tympanic cavity in two patients (Table 1). In three of the five cases, there was no significant difference between the choice of surgical approach based on imaging evaluation and on AR; however, in two cases (Cases 2 and 3), the choice changed significantly (Figure 2, *p* < 0.05). One patient (Case 2) had a slight mastoid extension (Figure 3a–c); therefore, 60% of the physicians thought they would adopt TEES. However, after seeing the AR images, 70% of the physicians thought they would adopt canal wall-up mastoidectomy (MES) without hesitation because it was considered to be more extensive than EAC drilling, judging from the AR images (Figure 3d,e). In the other case (Case 3), the antrum was filled with cholesteatoma (Figure 4a–c); therefore, 100% of the physicians thought that they would adopt MES. However, the extent of EAC drilling was not so large, judging from the AR images (Figure 4d,e), that 70% of the physicians thought they would adopt TEES. The cholesteatoma could be removed by TEES, and the ear canal defect was reconstructed using a single piece of tragal cartilage (Figure 4f,g). No re-retraction pockets were observed 1 year postoperatively (Figure 4h).

The five patients, including two males and three females, were aged between 5 and 51 years. The type of cholesteatoma was pars flaccida in four patients and congenital in one patient. All cases were operated on without complications, including four treated with TEES and one treated with MES. The extent of lesion extension identified during surgery was AM in three patients and TAM in two patients.

T: tympanic cavity, A: attic, M: mastoid referred to by the European Academy of Otology and Neurotology/Japan Otological Society (EAONO/JOS) staging system [23]; TEES: trans-canal endoscopic ear surgery; MES: canal wall-up mastoidectomy using a microscope.

## 4. Discussion

Techniques for middle ear cholesteatomas can be broadly divided into transcanal retrograde and transcortical approaches. The former mostly uses an endoscope currently, which avoids a retro-auricular incision, although it is also applied in MES. The latter, on the other hand, often uses a microscope and can preserve the EAC in canal wall-up mastoidectomy. The indications for endoscopic ear surgery for cholesteatoma have expanded to cases with mastoid extension in addition to the traditional indications for the tympanic cavity and attic. However, not all mastoid extension cases can be treated with TEES. Moreover, it is not always easy to predict the extent of EAC drilling required to remove the lesion and decide which approach should be applied. This is also indicated by the results of the questionnaire survey conducted in this study. That is, the survey showed that different doctors made different decisions, even though they shared the same strategy.

We attempted to predict the appropriate removal range of the EAC to resect the lesion by projecting a lesion on an intraoperative endoscopic image using AR preoperatively. As a result, the AR display was used as a reference to proceed with the surgery, and in two of five cases, the use of AR changed the approach considered based on the imaging findings. If AR had not been used, the approach would have to be changed during surgery, unnecessarily destroying normal structures, such as the EAC, and requiring more surgical time. For example, in Case 2, the cholesteatoma was localized in the mastoid cavity on preoperative images and was considered an indication for transcanal attico-antorotomy using a powered device [8,24]. However, AR projection at the time of surgery revealed that the lesion was located deep in the antrum, and extensive posterior wall drilling of the EAC was required to remove the cholesteatoma. Therefore, a transcortical approach was selected at the beginning of the surgery, and the posterior wall of the EAC was preserved. In contrast, in Case 3, although the cholesteatoma was extensive on the images, it was confirmed that a wide osteotomy was unnecessary based on the AR image. Because the area of reconstruction was small (Figure 4f), the EAC defect was completely and easily reconstructed with a single piece of tragal cartilage (Figure 4g), with favorable postoperative findings (Figure 4h). The main reason for this difference is that the size of the attic and antrum vary from person to person. The extent of EAC removal may depend not only on its size but also on its positional relationship with other structures, such as the width and angle of the EAC.

Tympanoplasty, a reconstructive procedure of the eardrum and ossicles, necessitates precise and minimal removal of the affected areas to maintain hearing. Minimizing the extent of osteotomy is required to ensure reconstruction of the posterior wall of the EAC and the lateral wall of the attic for cholesteatoma treatment [10], but this is not easy with conventional tympanoplasty because the location of the target lesion is unclear during surgery. The integration of AR technology enables surgeons to objectively visualize lesions and select the most appropriate surgical technique during the surgery.

**Figure 5:**
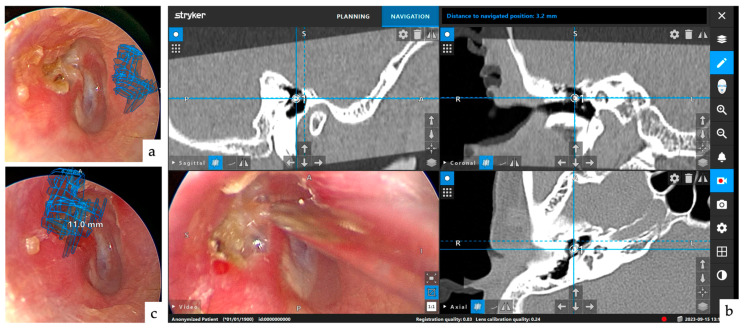
Correction of faulty registrations. (**a**) The lesion and augmented reality (AR) images (shapes in blue) do not match significantly. (**b**) Re-registration based on the lateral process of the malleolus. (**c**) After correction, the lesion and AR images match.

While imaging of the position of instruments matched with image data is a basic function of navigation systems in various fields, newer systems allow for additional features such as integrated elements of AR, image fusion, acoustic warning signals, and the use of intraoperative CT [25,26,27,28,29]. The application of AR in otorhinolaryngology, particularly in endoscopic sinus surgery (ESS), has been previously reported [30], and its role in MES for cochlear implantation has also been explored [31]. However, the use of AR in TEES and cholesteatoma has not been reported, making our study pioneering in this field. One concern regarding the use of navigation systems, including AR, in temporal bone surgery is their accuracy. Many structures, such as nerves and blood vessels, are located within the temporal bone, and their anatomy is such that they cannot be touched or observed until bone drilling, such as mastoidectomy, is performed. In addition, important organs, such as the cochlea, as well as vestibular and facial nerves, are densely located within a small area, with large individual differences in their movements; therefore, a highly accurate navigation system is required to ensure safety. The surface-matching registration method commonly used in sinus surgery is simple and noninvasive. However, its accuracy varies widely, with an error margin of approximately 2 mm [32]. In temporal bone surgery, changes in skin surface coordinates due to head torsion cause further variations in accuracy, and unacceptable levels of error are likely to occur in the deep temporal bone area. A paired-point method, in which a titanium screw is implanted in the bone surface in advance and CT scans are taken for registration, has been developed to improve accuracy. Although this method improves accuracy, it is psychologically and physically burdensome for patients because it requires an invasive procedure with patients in the awake state before surgery [33,34]. In this study, ENT navigation^TM^ (Striker, Kalamazoo, MI, USA), which allows for intraoperative modifications, was used when the surface-matching method did not provide sufficient accuracy. In cases where high accuracy was achieved at the surface but errors were noticeable at depths, such as in the tympanic cavity, high accuracy was achieved by re-registering the lateral process of the malleus as a reference (Figure 5a–c). The lateral process of the malleus was chosen as the reference because of its proximity to the site to be considered and because its position is unlikely to change with diseases or surgical manipulations. This modification was important because it allowed for reproducibility.

We have shown that a navigation system using AR can minimize osteotomies to preserve the normal structure in tympanoplasty, and objectively select the appropriate technique intraoperatively. To effectively use AR, proper preoperative diagnosis by CT/MRI is important, especially for the accurate evaluation of the range of cholesteatoma extension from the attic to the mastoid cavity to select the appropriate surgical approach. In order to accurately evaluate images, it is necessary to predict the extent of mastoid cavity extension based on the magnification of the mastoid cavity opening [35], the prediction by the temporal subtraction method [36], and the prediction of mastoid cavity extension by artificial intelligence (AI) [37]. These are based on CT images. However, despite the issues of the costs of MRI facilities and imaging, MRI is very useful [38,39].

**Figure 6:**
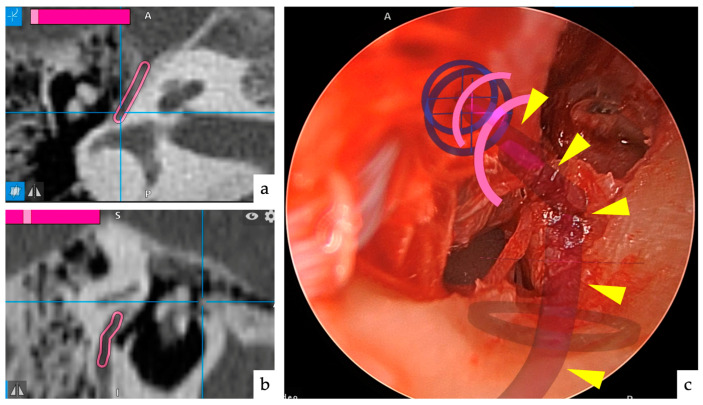
Augmented reality (AR) image of the facial nerve. (**a**) Marking the facial nerve with a pink line on the axial computed tomography (CT) slice of the temporal bone. (**b**) Marking the facial nerve with a pink line on the sagittal CT slice of the temporal bone. (**c**) An AR image of the facial nerve overlaid on the endoscopic image (yellow arrowhead tip). It is not a 3D display; therefore, its depth is not known, but its direction is indicated.

Despite the potential of AR to be applied to any temporal bone surgery, a challenge exists in AR-assisted surgery, which is the lack of three-dimensional features. Although AR is effective in identifying the site of the lesion, it does not reveal the depth of normal structures that require attention, such as facial nerves and blood vessels, and thus, the full potential of AR cannot be exploited (Figure 6a–c). However, because endoscopic images are originally 2D and their orientation can be determined, they may be of great value. Therefore, we believe that when robotic surgery is introduced in otology in the future, it will be performed with AR assistance. This is because robotic surgery, which has difficulty utilizing the surgeon’s hand sensations, requires more information to be obtained from the monitor.

Other challenges are the time required and the cost. The navigation unit is expensive, and peripheral equipment must also be purchased. CT must be used more extensively than usual for navigation, although special imaging conditions are not required to use AR images. Extra time is required to preoperatively plot the lesion, although this can be done in 10–15 min once the doctor is accustomed to the process. In addition to the usual registration of the probe, a registration of the endoscope must also be performed. Therefore, at this time, AR is used for limited cases; however, once the process is further simplified, it will be used for all cases.

This study has some limitations. First, the sample size was small (five patients) and included both pediatric and adult patients with different types of cholesteatoma. To make a universal statement, we must increase the number of cases studied. The pathogenesis of cholesteatoma varies widely, and there are naturally significant differences between congenital and acquired lesions. However, for the following reasons, we decided to present this report in its developing state: We had intended to report our findings after having examined many more cases; however, early on, we encountered a case in which the preoperative prediction and the actual surgical technique differed. The use of AR is promising, and there are indeed cases that differ from preoperative predictions. We believe that the difference will not be significant, regardless of the number of cases we include to demonstrate these points. Even if the pathology and etiology differ, the goal of complete removal of the lesion while preserving as much of the normal structure as possible remains (although the method of reconstruction may differ). In this study, we focused on cases that could benefit from TEES; we believe that it is problematic when extensive bone resection or posterior auricular incision is required despite TEES being scheduled. Second, ideally, the surgical approach should be determined using AR during the outpatient consultation phase in the process of surgical scheduling, which is, however, impossible because this is a navigation-based technique and registration is required. Third, this study was based on the assumption that our surgical strategy was the best option. Despite the variety of surgical techniques available, this report was limited to canal wall-up (TEES + transcortical mastoidectomy) or TEES with minimal EAC drilling. Although these should prove to be the best, they are difficult to achieve. As previous reports of surgical results show, the pathophysiology of cholesteatoma is so diverse that it is difficult to single out one strategy as the “best” one. The main goal in the treatment of cholesteatoma is to reduce the recurrence rate. Although residual disease has been shown to decrease with the use of endoscopes [40], recurrent disease remains a problem [2,3,4,5]. Thus, the idea of more reliable reconstruction is certainly an important strategy. It would be natural to aim for reliable resection of the lesion while preserving as much normal structure as possible for this purpose.

## 5. Conclusions

We report for the first time the use of AR imaging in TEES and show that AR-assisted surgery, which can minimize EAC drilling, is useful in TEES and facilitates the intraoperative selection of the most suitable technique. Although the number of cases was small in this study, we demonstrated that AR may change the approach considered preoperatively based on imaging findings. Further studies should be conducted to find a more reliable and simple registration method for better imaging of structures that require attention. Although there are still many issues to be addressed before AR can be used as a standard, this is an area where further growth is expected.

## Figures and Tables

**Table 1:** Characteristics of the five cases using augmented reality (AR).

Case	Age	Sex	Type	Range	TEES/MES
1	44	Male	pars flaccida	AM	TEES
2	51	Female	pars flaccida	TAM	MES
3	41	Male	pars flaccida	AM	TEES
4	5	Female	congenital	TAM	TEES
5	41	Male	pars flaccida	AM	TEES

## Data Availability

Data are available upon reasonable request to the corresponding authors.
